# Methionine at the Heart of Anabolism and Signaling: Perspectives From Budding Yeast

**DOI:** 10.3389/fmicb.2019.02624

**Published:** 2019-11-15

**Authors:** Adhish S. Walvekar, Sunil Laxman

**Affiliations:** Regulation of Cell Fate, Institute for Stem Cell Science and Regenerative Medicine (inStem), Bangalore, India

**Keywords:** methionine, S-adenosyl methionine, cell fate decisions, saccharomyces, metabolism, pentose phosphate pathway, NADPH, reductive biosynthesis

## Abstract

Studies using a fungal model, *Saccharomyces cerevisiae*, have been instrumental in advancing our understanding of sulfur metabolism in eukaryotes. Sulfur metabolites, particularly methionine and its derivatives, induce anabolic programs in yeast, and drive various processes integral to metabolism (one-carbon metabolism, nucleotide synthesis, and redox balance). Thereby, methionine also connects these processes with autophagy and epigenetic regulation. The direct involvement of methionine-derived metabolites in diverse chemistries such as transsulfuration and methylation reactions comes from the elegant positioning and safe handling of sulfur through these molecules. In this mini-review, we highlight studies from yeast that reveal how this amino acid holds a unique position in both metabolism and cell signaling, and illustrate cell fate decisions that methionine governs. We further discuss the interconnections between sulfur and NADPH metabolism, and highlight critical nodes around methionine metabolism that are promising for antifungal drug development.

## Introduction

**Figure 1:**
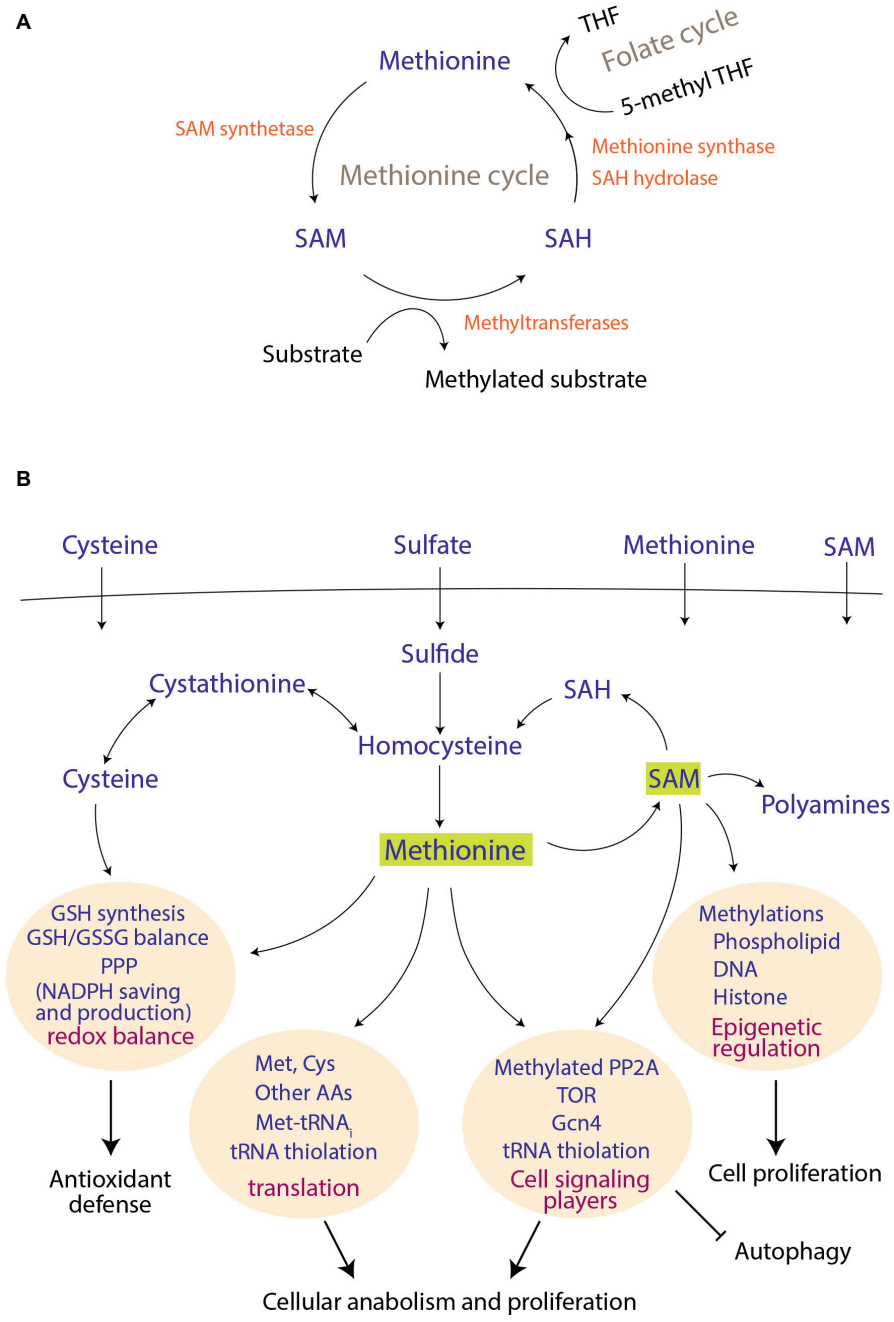
Methionine metabolism and its connections to cell signaling and proliferation outcomes. **(A)** Connections of the methionine cycle with one carbon metabolism. SAM, S-adenosyl methionine; SAH, S-adenosyl homocysteine; THF, tetrahydrofolate. **(B)** Uptake of sulfur metabolites, their assimilation and utilization. The coupling of methionine and its derivatives to different cellular processes and thereby to final cellular outcomes are shown.

For most researchers, methionine is invariably connected to the start of protein translation, as it is typically the first amino acid encoded in a polypeptide chain. However, this metabolite is biochemically unique among the 20 natural amino acids. Only methionine and cysteine have sulfur in their side-chains. While cysteine has a reactive thiol group, which affects redox balance and causes toxicity at higher concentrations (Deshpande et al., 2017), the sulfur group in methionine is uniquely protected, making it redox insensitive. Additionally, the masking of sulfur in methionine is elegantly used for one more function, which is the transfer of a methyl group. Suitable conversions of methionine to its derivatives [primarily S-adenosyl methionine (SAM)], and their connections to key metabolic and signaling pathways show that the role of methionine is not limited to the initiation of translation alone (Figures 1A,B). This mini-review focuses on the role of methionine as an anabolic signal.

Several studies using different model organisms suggest a role for methionine as a potent, universal growth cue. Among those, studies using *Saccharomyces cerevisiae* have revealed conserved facets of methionine-mediated effects, such as the inhibition of autophagy (Wu and Tu, 2011; Sutter et al., 2013), the regulation of tRNA thiolation, which controls overall metabolic state (Laxman et al., 2013; Gupta et al., 2019), and increased cell proliferation through the activation of conserved signaling pathways (Sutter et al., 2013; Walvekar et al., 2018). In mammals, several tumors need methionine for their survival and metastasis (Sugimura et al., 1959; Halpern et al., 1974; Breillout et al., 1990; Komninou et al., 2006; Mehrmohamadi et al., 2016; Gao et al., 2019; Sanderson et al., 2019). Studies using *Drosophila* that have been fed a high-methionine diet show that methionine increases their fecundity and growth (Troen et al., 2007), and its restriction correlates with increased lifespan (Lee et al., 2014, 2016). These studies suggest that methionine availability controls cell growth and determines cell fates. In the subsequent sections, we summarize important characteristics of methionine and methionine-derived metabolites, and highlight its roles in signaling and metabolism, as well as in translation (beyond being the first amino acid encoded in a polypeptide). We particularly emphasize the metabolic costs incurred during the biosynthesis of methionine and related metabolites, which suggest deeper interconnections with NADPH metabolism and the reductive biosynthetic capacity of a cell.

## The Biosynthesis of Methionine, Its Inter-conversion to Other Metabolites, and the Metabolic Pathways Involved

Although methionine is an essential amino acid (i.e., needs to be supplemented) for mammals, most fungi efficiently assimilate sulfates for methionine biosynthesis (Figure 1). Through a series of enzymatic steps, fungi reduce sulfate to sulfite, and finally to sulfide. Notably, each round of this reduction (to sulfide) consumes two ATP molecules, as well as reducing equivalents from four NADPH molecules [details reviewed in (Thomas and Surdin-Kerjan, 1997)]. This synthesis of homocysteine from sulfide marks sulfur incorporation into a carbon skeleton, and is a precursor for methionine as well as cysteine biosynthesis in yeast and other fungi (Figure 1B). The methyl group in methionine originates from 5-methyl tetrahydrofolate (5-methyl THF), a central metabolite in one-carbon metabolism (the folate cycle) (Figure 1A). Notably, the synthesis of 5-methyl THF itself also requires a further investment of NADPH, collectively revealing that this process of making methionine is perhaps the largest sink for NADPH utilization. In the case of cysteine biosynthesis, the transsulfuration pathway in yeasts converts homocysteine to cysteine through cystathionine (Cherest et al., 1993). In an evolutionary distinction, mammalian cells typically operate unidirectionally, i.e., they can convert homocysteine to cysteine, but not the other way round (Finkelstein, 1990). Yeasts, however, can directly utilize these two amino acids as a sole sulfur source for their growth (Thomas and Surdin-Kerjan, 1997).

S-adenosyl methionine (SAM), which is a universal methyl group donor, is perhaps the most important methionine-derivative. When the methyl group of SAM is transferred to different accepters, SAM is converted to S-adenosyl homocysteine (SAH), which can be subsequently converted to homocysteine and finally to methionine again, completing the cycle (Figure 1A). Thus, the methionine/SAM-, transsulfuration reactions-, and folate-cycles are closely interconnected, and the abundance of methionine/cysteine is reflected in increased SAM levels (Sutter et al., 2013; Laxman et al., 2014; Deshpande et al., 2017). Finally, methionine indirectly supports the synthesis of two other important molecules, i.e., glutathione (GSH) and polyamines. Cysteine is directly incorporated into the backbone of GSH, and SAM is required for the synthesis of polyamines (Figure 1B). Since all these metabolites play crucial roles in the maintenance of cellular homeostasis, and are critical for growth, they are therefore acutely sensed and trigger signaling responses, as described subsequently.

## Methionine Sensing/Signaling and Its Role in Regulating Translation With Metabolism

### Sensing Methionine: Connections to Translation

Although methionine and other amino acids are transported across the plasma membrane by broad-specificity transporters, upon entry into the cell, there seem to be specific sensing mechanisms that trigger downstream events. Methionine is quickly converted to SAM in yeast (Laxman et al., 2013, 2014; Deshpande et al., 2017), and the amounts of SAM appear to be central to methionine sensing. In yeast, at least two known (and evolutionarily conserved) mechanisms gauge methionine availability. The addition of methionine immediately increases the amounts of specific, conserved thiol-modifications on tRNAs (tRNA U34 thiolation) (Laxman et al., 2013). These act as methionine sensing systems that control metabolic outputs (Gupta et al., 2019). Methionine is connected to translation in other ways as well, apart from its role as an initiator-amino acid and in the tRNA thiolation pathway. Specific SAM-dependent methyltransferases activate the master-regulator of growth and translation [the Target of Rapamycin (TORC1) pathway] by controlling upstream regulators of TORC1 (Sutter et al., 2013). Additionally, another SAM sensor called SAMTOR (which regulates mTOR) is present in mammalian systems (Gu et al., 2017). This methionine-dependent activation of TORC1 increases overall translation capacity (through increased ribosome synthesis, which is the canonical output of TORC1 activity). A recent study showed that the presence of excess methionine also induces the transcription of ribosomal and rRNA genes, and therefore overall translation capacity (Walvekar et al., 2018). While the mechanism is not fully known, this might also couple to the activation of TORC1 by methionine (Sutter et al., 2013). Overall, methionine abundance supports increased translation through many underappreciated roles beyond initiating polypeptide synthesis: this includes increased TORC1 activity, the control of metabolism and growth by a tRNA modification, and increased amounts of ribosomal transcripts, proteins, and rRNAs.

### Methionine and Metabolism

While methionine availability and growth have been long studied, especially in mammalian cells (Eagle, 1959; Finkelstein, 1990), a recent study in yeast directly showed that methionine acts as an anabolic signal, and regulates metabolic transformation (Walvekar et al., 2018). This study identifies the core metabolic program that methionine controls, and was built on long standing observations in yeast that the presence of methionine increases growth and inhibits autophagy (Wu and Tu, 2011; Sutter et al., 2013; Laxman et al., 2014). The presence of excess methionine results in global gene expression changes, with a unique anabolic transformation signature (Walvekar et al., 2018). In this anabolic program, the host of genes regulated by methionine that were involved in metabolism could be organized into a clear hierarchy. In this hierarchy, methionine simultaneously upregulates genes involved in three key metabolic nodes: the pentose phosphate pathway (PPP), glutamate synthesis, and pyridoxal phosphate synthesis. The metabolites and co-factors produced in these three nodes were central for the function of a set of metabolic proteins, which itself were transcriptionally induced by methionine. Notably, these proteins were involved in the synthesis of nearly all other amino acids, as well as nucleotides. Essentially, this study demonstrated that the presence of methionine (even during the limitation of other amino acids) triggers the synthesis of all other amino acids and nucleotides, required to sustain anabolism. The very core of this anabolic program relies on the production of metabolites and co-factors that enable reductive biosynthesis (Walvekar et al., 2018). Consistent with the observations in this study, the methionine-sensing tRNA thiolation modification pathway also results in a global metabolic rewiring by regulating flux through the PPP (unexpectedly by controlling phosphate availability) (Gupta et al., 2019). In methionine replete condition, cells without tRNA thiolation divert the carbon flux toward synthesis of trehalose, thereby releasing and recycling the trapped phosphate. By doing so, methionine availability ensures balance of carbon and nitrogen metabolism in cells. The role of high PPP activity in growth is now widely established. However, this underappreciated connection of methionine to the PPP was first made in yeast (Thomas et al., 1991; Campbell et al., 2016), and the more recent studies provide an overarching picture of the nature of metabolic regulation by methionine (Walvekar et al., 2018; Gupta et al., 2019). Finally, there is a long-known coupling of methionine metabolism with one-carbon folate cycle, which appears to be universal (Thomas and Surdin-Kerjan, 1997; Locasale, 2013). Collectively, studies in yeast have revealed that methionine is perceived as a strong growth signal, and triggers an organized anabolic program in cells.

### Sensing Methionine: Connections to TOR and Autophagy

One of the first observations that the major growth regulating pathway in eukaryotes, the TORC1 pathway, responds to methionine were made in yeast. Studies in *S. cerevisiae* identified an acute induction of autophagy in cells that were exclusively methionine limited (but not general amino acid starved) (Wu and Tu, 2011). Subsequent studies showed that methionine activates the TOR pathway through the SAM-mediated methylation of PP2A (Sutter et al., 2013). Methylated PP2A promotes the de-phosphorylation of a major TORC1 negative regulator, Npr2, which itself inhibits TOR and translation, but activates autophagy. By de-phosphorylating Npr2, methionine inhibits the Iml1p/Npr2p/Npr3p complex, and therefore releases cells from autophagy. Separately, the methionine sensing tRNA thiolation pathway also appears to be coupled to TORC1 (Laxman and Tu, 2011; Candiracci et al., 2019), although the mechanisms are yet to be found. Thus, cells sense methionine availability through multiple systems, reflecting the importance of this molecule. Finally, among the myriads of methylation events that SAM supports, phospholipid and histone methylation are the major consumers of methyl groups, and thereby connect methionine/SAM levels to epigenetic regulation (Ye et al., 2017). This process is also dependent on the SAM dependent methylation of PP2A (Sutter et al., 2013), suggesting this deep coupling of methionine/SAM to TORC1, PP2A, and signaling.

## Methionine as a Signal for Growth

In summarizing the earlier section, we note that cell fate decisions are complex processes, where different nutrient inputs are weighed against the cellular needs, to determine an outcome. Sentinel metabolites, such as acetyl CoA (Cai et al., 2011; Pietrocola et al., 2015; Krishna and Laxman, 2018), play critical roles in these decisions, particularly in commitments toward cell growth. Here, methionine has emerged as one such sentinel metabolite. As discussed earlier, observations made first in *S. cerevisiae* revealed that methionine abundance (even during overall amino acid limitation) can inhibit autophagy (Sutter et al., 2013), and increases cellular anabolism and proliferation (Walvekar et al., 2018). This proliferative response is striking as it is observed even in the absence of other free amino acids, and the addition of methionine alone results in cells inducing a strong anabolic program (Walvekar et al., 2018). This general principle of methionine acting as a growth signal appears to be conserved across different organisms. Indeed, several tumors need methionine for their survival and metastasis (Sugimura et al., 1959; Halpern et al., 1974; Breillout et al., 1990; Gao et al., 2019). Methionine supplementation in *Drosophila* results in increased fecundity and growth (Troen et al., 2007). Methionine restriction, on the other hand, is implicated in the increased lifespan of *Drosophila* (Lee et al., 2014, 2016) as well as other animal models (Orentreich et al., 1993). These studies argue for the unique position that methionine holds in cell fate determination, as a potent growth cue.

## Connection of Methionine Metabolism to NADPH

Finally, we speculate on why methionine might be such a potent growth cue, by reiterating its close coupling to NADPH and the reductive capacity of a cell. As described earlier, the reduction of sulfate to sulfide, and its incorporation of sulfur in the carbon skeleton is an energy intensive process. The *de novo* biosynthesis of one molecule of methionine requires an investment of at least six molecules of NADPH molecules (Thomas and Surdin-Kerjan, 1997; Kaleta et al., 2013). Therefore, methionine (along with cysteine) is perhaps the costliest amino acid to be biosynthesized. Given this extremely large reductive cost to methionine synthesis, cells appear to gauge methionine availability as an indicator of NADPH availability, and of overall reductive biosynthetic capacity. This is reflected in the close coupling of methionine availability with (notably) the PPP activity (Campbell et al., 2016; Walvekar et al., 2018). The PPP, in addition to making ribose sugars, is the major generator of NADPH in cells (Chen et al., 2019). This connection of the PPP with methionine is exemplified in the *ZWF1* null mutant in yeast. The *ZWF1* gene in *Saccharomyces cerevisiae* encodes for glucose-6-phosphate dehydrogenase, the first enzyme in the PPP. It is also one of the two steps in PPP where NADPH is generated. The *ZWF1* null mutant is a methionine auxotroph (Thomas et al., 1991), and shows decreased tolerance to oxidants, even in the presence of methionine, indicating that NADPH balance in the presence of methionine is critical (Campbell et al., 2016). The most direct connection was revealed in a recent study, which showed that in methionine excess, transcripts of PPP genes were strongly induced (Walvekar et al., 2018). Separately, the methionine-sensing tRNA thiolation pathway also controls flux through the PPP in tune with methionine availability (Gupta et al., 2019). Collectively, these data from yeast show that methionine while strongly drive anabolic processes, all these are closely tied to NADPH synthesis and utilization. In summary, methionine availability appears to be a very good indicator of reductive biosynthetic capacity in cells.

## Targeting Methionine Metabolism or Sensors for Novel Antifungals

**Figure 2:**
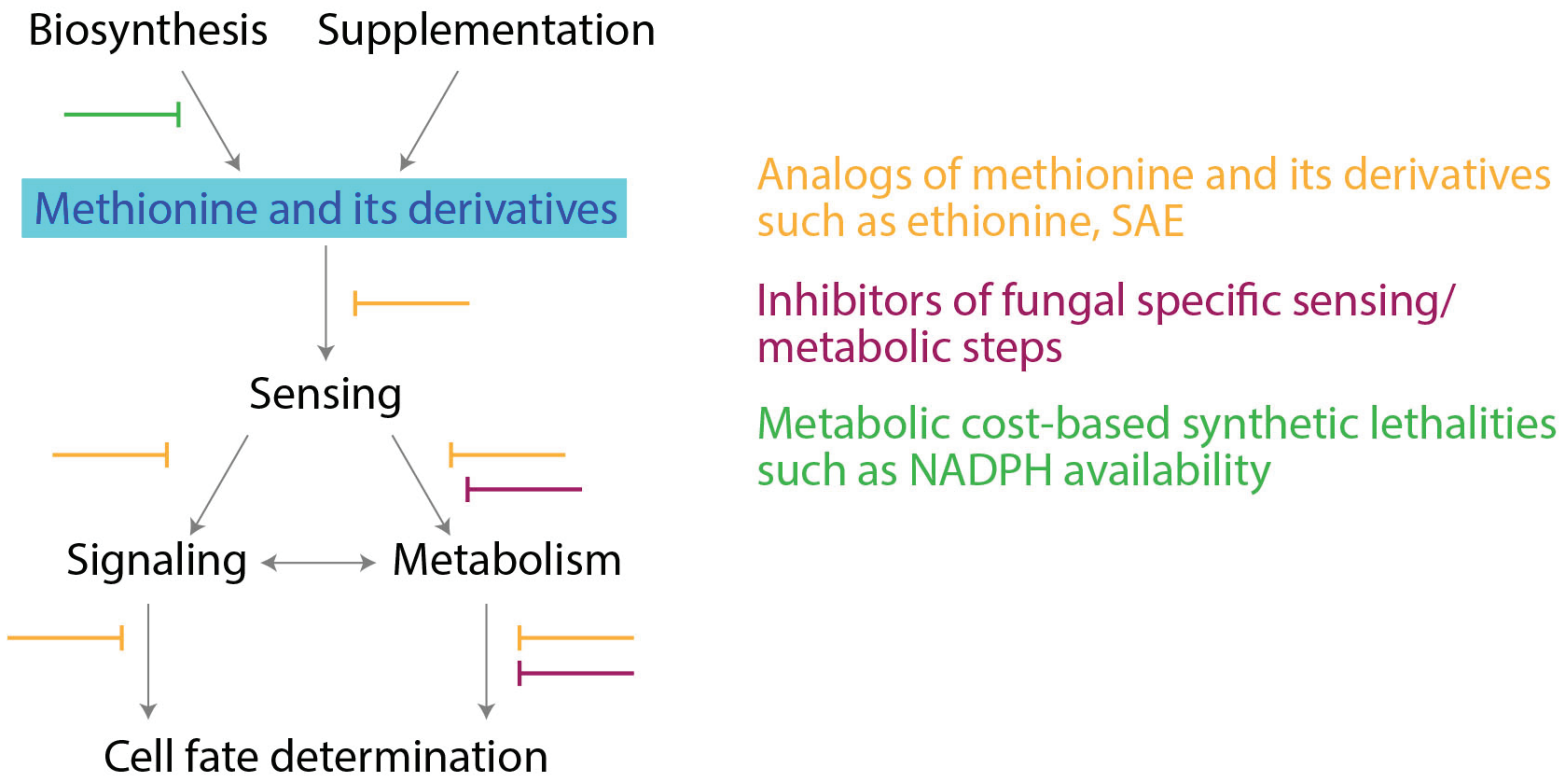
Targeting methionine metabolism for antifungal drug development. Potential strategies for development of antifungal drugs, along with the critical nodes in methionine metabolism are shown.

The key position that methionine metabolism holds, suggests opportunities to develop novel antifungal drugs (summarized in Figure 2). Notably, most fungi need to assimilate sulfur and biosynthesize methionine/sulfur metabolites from sulfates. The *de novo* biosynthesis of methionine is essential in *Magnaporthe oryzae* (rice blast fungus) for effective infection (Saint-Macary et al., 2015). L-Homocysteine accumulation after inhibition of methionine biosynthesis affects ergosterol biosynthesis (Jastrzębowska and Gabriel, 2015), and as discussed earlier, *ZWF1* null mutants are methionine-auxotrophs in yeast. Therefore, inhibition at both levels (the synthesis of methionine and the PPP), could be lethal for fungal growth and pathogenesis. Indeed, in the major fungal pathogen *Candida albicans*, methionine uptake and its metabolism to polyamines is required for the yeast to mycelial transition, a critical step during pathogenesis, as well as for virulence (Schrevens et al., 2018). Therefore, could the inhibition of critical steps in methionine metabolism potentially block fungal proliferation and virulence? Notably, mammalian cells do not assimilate and reduce sulfur, and rely instead entirely on supplied methionine. Hence, while the effects of novel inhibitors of methionine biosynthesis (sulfate reduction), or specific fungal methyltransferases, could be minimal in mammals, this could allow selective fungal inhibition. An alternate strategy could be to use redesigned methionine analogs, such as ethionine. Ethionine has shown anticancerous/antiproliferative activity (Levy et al., 1953). Combining ethionine with a fungal specific inhibitor of biosynthesis of methionine, could be effective in inhibiting fungal growth and metabolism. Some of these ideas are illustrated in Figure 2.

## Conclusions

Studies using budding yeast have been instrumental in understanding the role of methionine as a potent growth cue. These studies have revealed intimate connections of methionine to metabolic control, signaling, and translation (beyond merely the initiation of translation). These are all exciting areas of basic research, and studies on yeasts are likely to uncover more secrets of how methionine is sensed, and how it controls metabolism and growth. Importantly, the previous studies have revealed a critical dependence of several fungi on methionine and its metabolites, suggesting a possible node to develop novel antifungal drugs.

## Author Contributions

All authors listed have made a substantial, direct and intellectual contribution to the work, and approved it for publication.

### Conflict of Interest

The authors declare that the research was conducted in the absence of any commercial or financial relationships that could be construed as a potential conflict of interest.

## Abbreviations

SAM: S-Adenosyl methionine
SAH: S-Adenosyl homocysteine
THF: Tetrahydrofolate
PPP: Pentose phosphate pathway
GSH: glutathione.
